# CLE-10 from *Carpesium abrotanoides* L. Suppresses the Growth of Human Breast Cancer Cells (MDA-MB-231) In Vitro by Inducing Apoptosis and Pro-Death Autophagy Via the PI3K/Akt/mTOR Signaling Pathway

**DOI:** 10.3390/molecules24061091

**Published:** 2019-03-20

**Authors:** Li Tian, Fan Cheng, Lei Wang, Wen Qin, Kun Zou, Jianfeng Chen

**Affiliations:** Hubei Key Laboratory of Natural Products Research and Development, China Three Gorges University, Yichang 443002, China; litian0401@126.com (L.T.); fancy1351@163.com (F.C.); wang-lei1989@hotmail.com (L.W.); shmily900920@163.com (W.Q.)

**Keywords:** autophagy, apoptosis, PI3K/AKT/mTOR, CLE-10, LC3, MDA-MB-231

## Abstract

Background: The antitumor activity of CLE-10 (4-epi-isoinuviscolide), a sesquiterpene lactone compound, isolated from *Carpesium abrotanoides* L. has rarely been reported. The aim of this study is to investigate the antitumor activity of CLE-10 and give a greater explanation of its underlying mechanisms. Methods: The cytotoxicity of CLE-10 was evaluated using MTT assay. Autophagy was detected by the formation of mRFP-GFP-LC3 fluorescence puncta and observed using transmission electron microscopy, while flow cytometry was employed to detect apoptosis. The protein expressions were detected through Western blotting. Results: CLE-10 induced pro-death autophagy and apoptosis in MDA-MB-231 cells by increasing the protein expression of LC3-II, p-ULK1, Bax, and Bad, as well as downregulating p-PI3K, p-Akt, p-mTOR, p62, LC3-I, Bcl-2, and Bcl-xl. CLE-10 that was pretreated with 3-methyladenine (3-MA) or chloroquine (CQ) weakened the upregulation of the protein expression of p-ULK1, or the downregulation of p62, p-mTOR, and decreased the level of cytotoxicity against MDA-MB-231 cells. Meanwhile, rapamycin enhanced the effect of CLE-10 on the expression of autophagy-related protein and its cytotoxicity, with the IC_50_ value of CLE-10 decreasing from 4.07 µM to 2.38 µM. Conclusion: CLE-10 induced pro-death autophagy and apoptosis in MDA-MB-231 cells by upregulating the protein expressions of LC3-II, p-ULK1, Bax, and Bad and downregulating p-PI3K, p-Akt, p-mTOR, p62, Bcl-2, and Bcl-xl.

## 1. Introduction

Breast cancer is one of the most prevalent cancers diagnosed in women worldwide, causing more than 500,000 deaths every year, especially in more developed regions [1]. Breast cancer treatment has developed from single surgery to multidisciplinary treatment including radiotherapy, chemotherapy, and endocrine therapy, significantly improving the prognosis of breast cancer [2]. However, the cancer often metastasizes or recurs because of drug resistance and toxicity [3]. Therefore, it benefits public health to explore novel anti-breast cancer agents.

Aiming at apoptosis and autophagy, two different types of programmed cell death (PCD) with their own distinctive features are significant in cancer chemotherapy. Apoptosis, triggered by extrinsically or mitochondria-mediated pathways, is a crucial cytotoxic mechanism of anticancer agents [4]. Moreover, Bcl-2 family proteins are involved in the mitochondria-mediated pathways. Autophagy is a highly conserved cellular activity during which cytoplasmic components including organelles or proteins are degraded and recycled [5]. The basal level of autophagy is usually appeared under certain stimuli or stress, contributing to the maintenance of normal cellular homeostasis. In that circumstance, autophagy acts to promote cell survival [6]. Recent researches have indicated that multiple anticancer treatments lead to excessive activation of autophagy and further result in cancer cell death [7]. In the process of autophagy, the soluble cytoplasmic form of LC3-I is transformed into its membrane associated form LC3-II, involved in the formation of autophagosomes, and finally degraded by autolysosomes [8]. Therefore, it is essential to calculate the amount of LC3-II degraded by lysosomes by comparing LC3-II levels with or without lysosomal protease inhibitor. Sequestosome1 (p62/SQSTM 1), acting as a vital adaptor of target cargo in the process of autophagy, also interacts with other proteins related to autophagy such as LC3 and beclin1 [9,10].

Accumulated evidence has revealed that the activation of the PI3K/Akt/mTOR signaling pathway leads to the occurrence of malignant tumors, indicating that the targeted suppression of certain components in this pathway might be a potential therapeutic strategy for cancer treatment [11,12]. Class1 I PI3K is a heterodimer of the p85 and p110 subunits with dual activities of lipid kinases and protein kinases. Class I PI3 K activates the serine/threonine kinase Akt, and Akt directly activates mTOR via mTORC1 at S2448 or indirectly through TSC2. The inactivation of TSC2 leads to the phosphorylation of Akt, which promotes cell survival by upregulating mTORC1 activity through cascaded signaling molecules to inhibit apoptosis and autophagy [13]. mTOR, acting as an autophagy inhibitor, prevents ULk1 activation and disrupts the mutual effect between ULk1 and AMPK. Conversely, ULK1 also suppresses mTOR by phosphorylation [14].

Natural products are the major resources for new cancer therapies. CLE-10, a sesquiterpene lactone compound, was obtained from *Carpesium abrotanoides* L. (CAL), a traditional Chinese herb, which has been employed to reduce fever or insect bites [15]. Moreover, a compound from the composite plant possesses antifungal, antioxidant, and cytotoxicity properties [16,17]. Studies have shown that CLE-10 isolated from *Inula britannica* or *Carpesium faberi* exhibits cytotoxic activity against several human cancer cells, and the IC_50_ value of CLE-10 on another breast cancer cell, MCF-7, was 45.97 ± 1.21 µM [18,19]. Although there is a wide interest in and extensive use of this medicinal herb, the underlying antitumor mechanism of CLE-10 is rarely reported. In this study, we found that CLE-10 inhibited the proliferation of breast cancer cells (MDA-MB-231) by inducing apoptosis and pro-death autophagy through the PI3K/Akt/mTOR signaling pathway.

## 2. Results

### 2.1. MTT Assay

**Figure 1:**
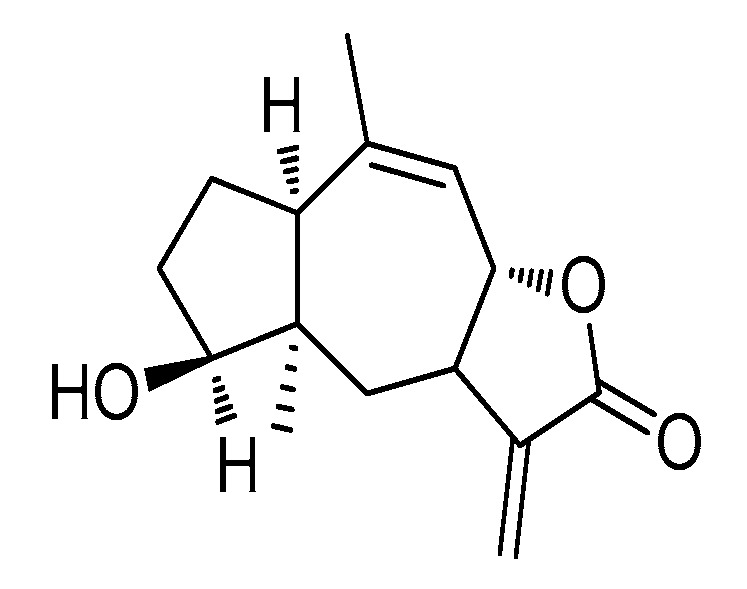
The chemical structure of CLE-10.

**Figure 2:**
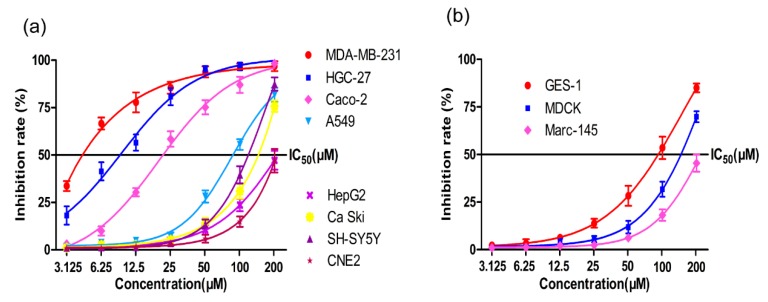
The cytotoxicity of CLE-10 on (**a**) MDA-MB-231, CaCo-2, A549, HepG-2, Caski, SH-SY5Y, HGC-27, CNE-2, (**b**) GES-1, MDCK, and Marc-145 by MTT assay. The data were representative results of three independent tests.

The structure of CLE-10 is presented in Figure 1 [20]. We investigated the cytotoxicity of CLE-10 on various cell lines. As described in Figure 2, MDA-MB-231 cells were the most sensitive to CLE-10 among the cell lines examined, with an IC_50_ value of 4.07 µM. In addition, CLE-10 showed lower cytotoxicity on normal cells (Figure 2b).

### 2.2. Inhibition of Autophagy Relieved CLE-10-Induced Cell Death

**Figure 3:**
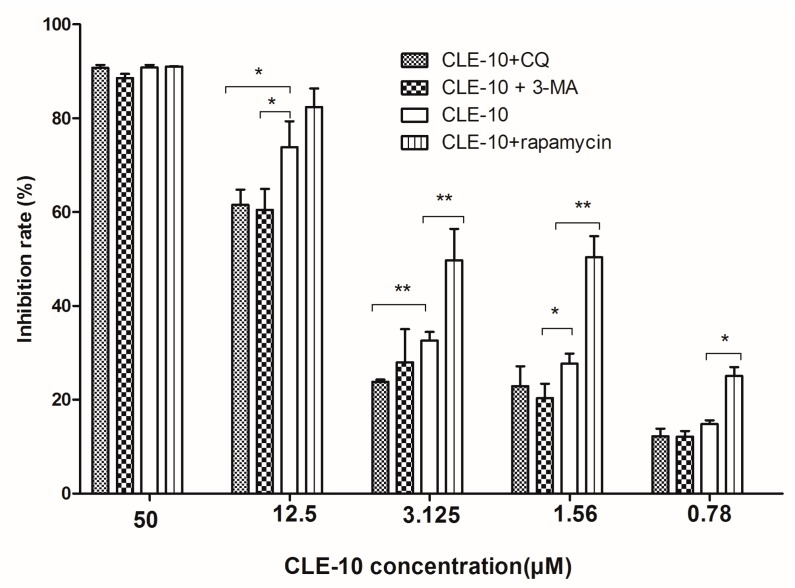
The inhibitory effect of CLE-10 pretreated with or without autophagy inhibitors (5 mM 3-methyladenine (3-MA), 20 µM chloroquine (CQ)) or inducer (100 nM rapamycin) on the proliferation and growth of MDA-MB-232 cells for 48 h. (* *p* < 0.05, ** *p* < 0.01, compared with the CLE-10 group).

To corroborate the impact of autophagy on CLE-10-induced MDA-MB-231 cell death, CLE-10 was used, pretreated with an autophagy inhibitor (chloroquine (CQ), 3-methyladenine (3-MA)) and a mTOR agonist (rapamycin). The concentration of inhibitors was at a safe level with no cytotoxicity against MDA-MB-231 cells. Autophagy inhibitors 3-MA or CQ weakened the inhibition of CLE-10 on the growth of MDA-MB-231 cells with the IC_50_ levels of 6.91 µM and 6.49 µM, respectively (Figure 3). There was no distinct difference on the inhibitory effect between the CLE-10 + 3-MA group and the CLE-10 + CQ group. Furthermore, rapamycin significantly enhanced the inhibitory effect of CLE-10, especially at a low CLE-10 concentration with the IC_50_ of 2.38 µM, indicating that CLE-10 induced autophagy, leading to breast cancer cell death rather than a protective mechanism.

### 2.3. CLE-10 Induced MDA-MB-231 Cell Apoptosis

**Figure 4:**
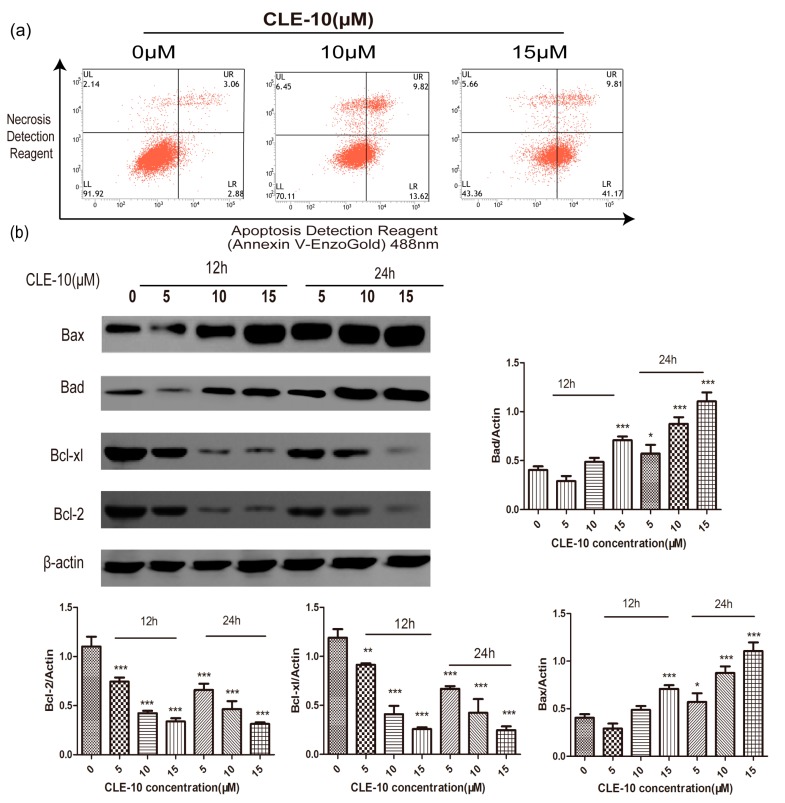
CLE-10 induced apoptosis in MDA-MB-231 cells. (**a**) Apoptosis induced by CLE-10 (0, 10, 15 µM) in MDA-MB-231 cells was detected by flow cytometry. (**b**) Representative Western blotting bands of Bcl-2, Bcl-xl, Bax, and Bad in MDA-MB-231 cells. Next to the bands are protein expression levels (* *p* < 0.05, ** *p* < 0.01, *** *p* < 0.001 compared with the 0 µM CLE-10 group).

The rates of apoptotis (accumulating both in Annexin V-Enzo Gold-positive/Necrosis Detection Reagent-negative (early apoptosis) and Annexin V-Enzo Gold-positive/Necrosis Detection Reagent-positive (late apoptosis)) were 5.94%, 23.44%, and 50.98% (Figure 4a,b) after treating with CLE-10 (0, 10, and 15 µM) for 24 h. These data indicated that CLE-10 exerted obvious apoptosis in a dose-dependent manner. In addition, Western blot revealed that CLE-10 downregulated Bcl-2 and Bcl-xl expressions and upregulated the expression of Bax and Bad (Figure 4b).

### 2.4. CLE-10 Induced MDA-MB-231 Cell Autophagy

**Figure 5:**
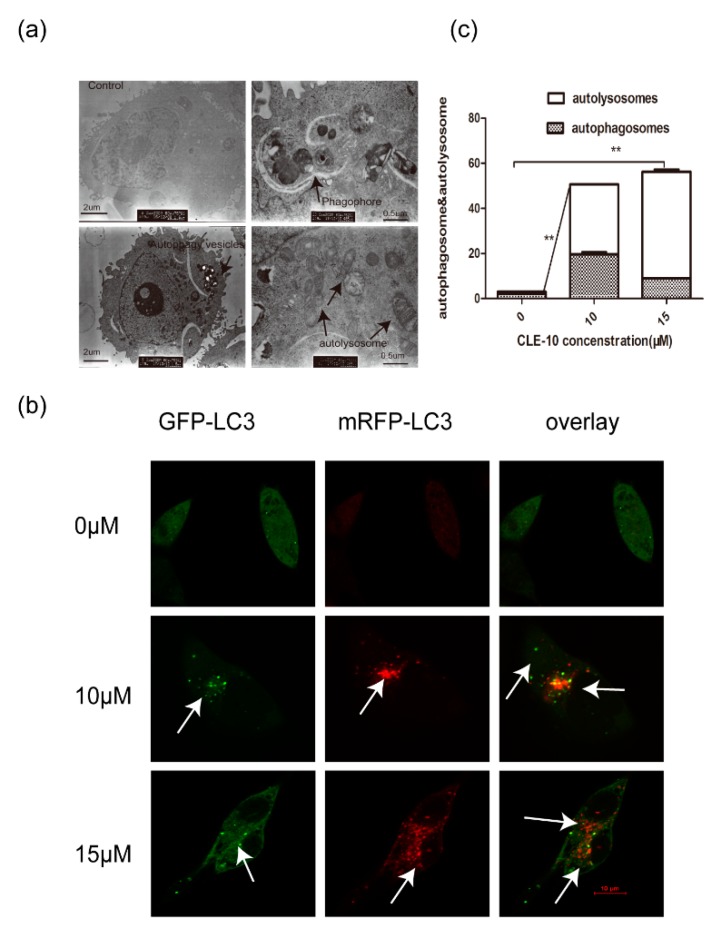
CLE-10-induced MDA-MB-231 cell death was mediated by autophagy. (**a**) Autophagy vesicles, autophagosomes, and autophagy lysosomes were observed in MDA-MB-231 cells after treatment with CLE-10. (**b**) MDA-MB-231 cells transfected with mRFP-GFP-LC3 adenovirus were detected with a confocal fluorescence microscopy. (**c**) The number of autophagosomes (yellow dots) and autolysosomes (red-only dots) in the control group and the CLE-10 group (** *p* < 0.01).

To confirm whether CLE-10 induced autophagy in MDA-MB-231 cells, TEM observation and the formation of mRFP-GFP-LC3 puncta were employed. Transmission electron microscopy indicated an enhanced presence of autophagosomes, autophagic vesicles, and autolysosomes in cells treated with 15 µM CLE-10 for 24 h (Figure 5a). Condensed cytoplasm, membrane invagination, and the disappearance of microvilli were observed at the same time. To further research the autophagy flux induced by CLE-10, MDA-MB-231 cells were transfected with mRFP-GFP-LC3 adenovirus before treatment with CLE-10. Autophagic flux was analyzed by the evaluation of mRFP-LC3 and GFP-LC3 puncta locations. GFP fluorescence (green dots), quenched easily in autolysosomes, is visible only in autophagosomes. Meanwhile, mRFP (red dots) signals can be observed under the environment of autophagosomes and autolysosomes. When the two colors merged together, yellow dots representing autophagosomes and red dots representing autolysosomes can be observed. As illustrated in Figure 5b,c, obvious enhancement in the amount of yellow puncta (autophagosomes) and red-only puncta (autolysosomes) appeared and the number of red dots was greater than the number of yellow dots, indicating that CLE-10 accelerated autophagic flux without suppressing the function of lysosomes or the fusion of autophagosomes and lysosomes. Therefore CLE-10 served as an autophagy inducer.

### 2.5. CLE-10 Inhibited the PI3K/Akt/mTOR Signaling Pathway in MDA-MB-231 Cells

**Figure 6:**
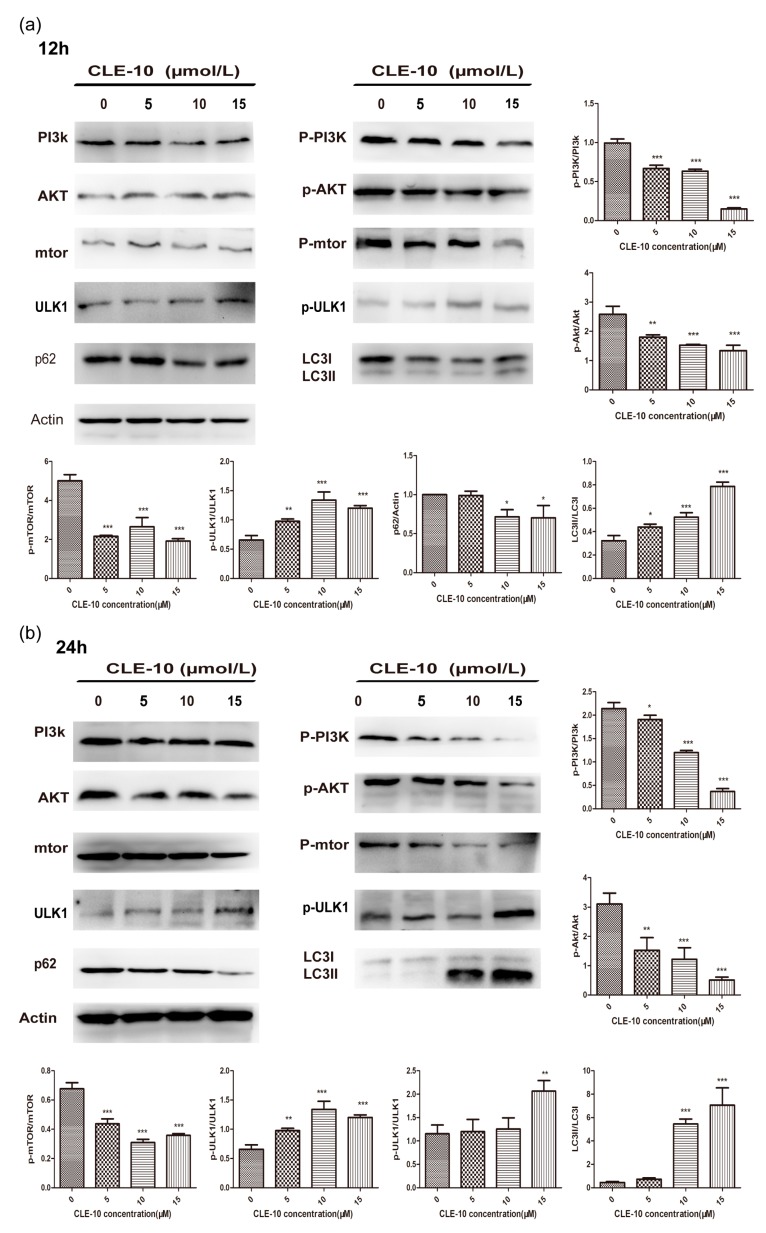
Influence of CLE-10 on the expression of the PI3K/Akt/mTOR signal pathway and autophagy-related proteins. (**a**) After 12 h of treatment with CLE-10, PI3K, Akt, mTOR, p-PI3K, p-Akt, p-mTOR, ULK1, p-ULK1, p62, LC3-I, and LC3-II expressions in MDA-MB-231 cells were analyzed by Western blot. (**b**) After 24 h of treatment of CLE-10, PI3K, Akt, mTOR, p-PI3K, p-Akt, p-mTOR, ULK1, p-ULK1, p62, LC3-I, amd LC3-II expressions in MDA-MB-231 cells were detected by Western blot. Data are expressed as the mean ± SD (*n* = 3). (* *p* < 0.05, ** *p* < 0.01, *** *p* < 0.001 compared with the 0 µM CLE-10 group).

The PI3K/Akt/mTOR signaling pathway is closely related to the enhancement of autophagy and is always activated in cancers, including breast cancer [21]. Therefore, we investigated whether the phosphorylation of PI3K, Akt, mTOR, and autophagy-related proteins (ULK1, p62, LC3) was involved in autophagy induced by CLE-10 in MDA-MB-231 cells. As shown in Figure 6, CLE-10 treatment for 12 h or 24 h in MDA-MB-231 cells downregulated PI3k, Akt, mTOR, and ULK1 phosphorylation in a dose-dependent manner but did not exert a significant effect on the expression of these total proteins. Meanwhile CLE-10 also reduced the protein expression of p62, LC3-I, and increased the protein expression of LC3-Ⅱ.

### 2.6. The Effect of CLE-10 Combined with 3-MA, CQ, or Rapamycin on the Protein Expression of mTOR, ULK1, p62, and LC3

**Figure 7:**
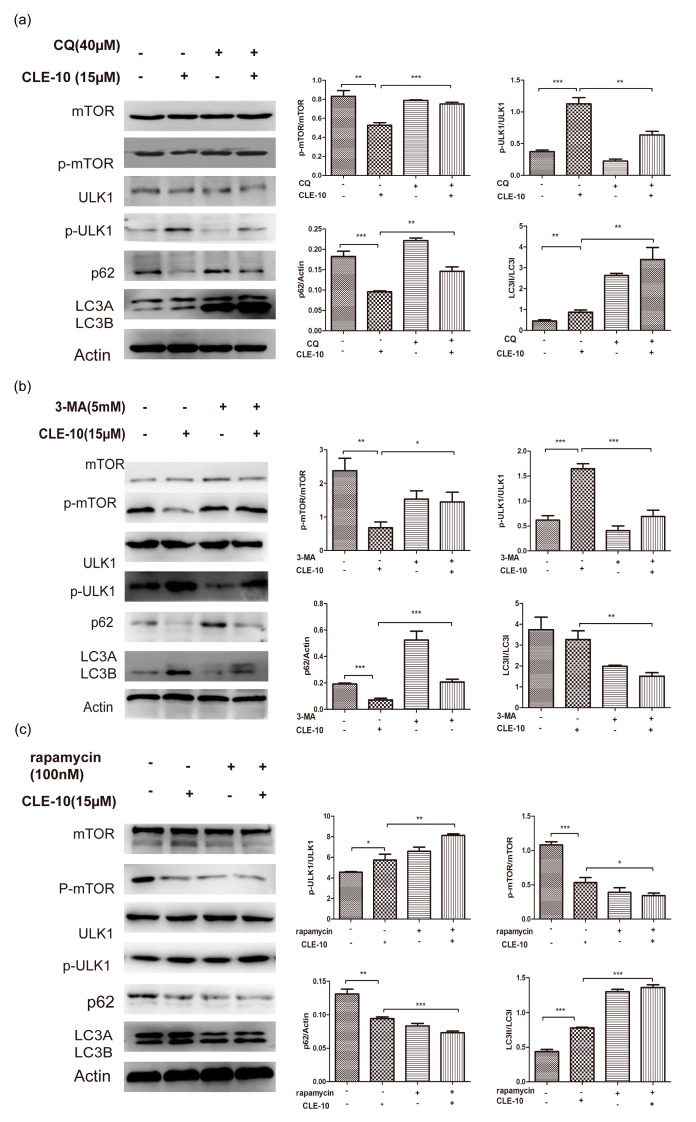
Effect of CLE-10 on autophagy-related proteins LC3-I/II, p-ULK1, ULk1, p62, mTOR, and p-mTOR were detected with and without autophagy inhibitor 3-MA, CQ, as well as the mTOR agonist rapamycin by Western blot analysis. (**a**) CLE-10 was used in combination with CQ. (**b**) CLE-10 was used in combination with 3-MA. (**c**) CLE-10 was used in combination with rapamycin. (* *p* < 0.05, ** *p* < 0.01, *** *p* < 0.001 compared with the control group or the CLE-10 group).

In addition, we detected autophagy-related proteins mTOR, p-mTOR, LC3, p-ULK1, ULK1, and p62 expression with and without autophagy inhibitors CQ, 3-MA, and mTOR inhibitor rapamycin by Western blot. As shown in Figure 7, the CLE-10, rapamycin, and CLE-10 + rapamycin treatments increased p-ULK1 and LC3-II expression as well as downregulated p62, p-mTOR, and LC3-І protein expression, with no obvious influence on the protein expression of mTOR and ULK1 as compared with the control group. The CLE-10 + 3-MA and CLE-10 + CQ treatments inhibited the upregulation of p-ULK1 expression and the downregulation of p-mTOR and p62 protein expression compared with the CLE-10 group. The CLE-10+3-MA treatment inhibited the protein expression of LC3-II while the CLE-10 + CQ treatment increased the protein expression of LC3-II compared with the CLE-10 group.

## 3. Discussion

The antitumor activity of CLE-10, a sesquiterpene lactone compound isolated from *Carpesium abrotanoides* L., has rarely been reported [19]. Therefore, we examined the cytotoxicity of CLE-10 and its underlying mechanisms. In this study, we demonstrated that CLE-10 had effective cytotoxicity against multiple human tumor cell lines tested, especially against MDA-MB-231 cells (IC_50_ = 4.07 µM). Meanwhile, CLE-10 showed lower cytotoxicity on normal cells, suggesting the selectivity of CLE-10 against tumor cells. Our results showed that CLE-10 induced apoptosis in MDA-MB-231 cells as evidenced by a growing number of apoptotic cells after flow cytometry (FCM) detection. This conclusion was further supported by increased expressions of Bax and Bad as well as downregulated Bcl-xl and Bcl-2 expressions. Bcl-2 family proteins are classified into three types. One type of protein inhibits apoptosis, such as Bcl-xl and Bcl-2, whereas a second type (Bak, Bax) accelerates apoptosis and another diverse type of BH3-only proteins (BIKn, Bad) can bind and control the anti-apoptotic Bcl-2 proteins to show the same function as the second type [22].

Meanwhile, CLE-10-induced MDA-MB-231 cell death and apoptosis was mediated by autophagy. TEM observation revealed significant morphological changes that were characteristic of autophagy such as membrane invagination and the existence of autophagic vacuoles and autolysosomes. Autophagy is an essential intracellular degradation process, during which lysosomes degrade and recycle cellular components, providing energy and new materials to maintain cellular environmental homeostasis [23]. The whole process of autophagy is called autophagy flux. Emerging evidence supports that impaired autophagic flux is related to a number of human diseases including tumorigenesis, cardiovascular system disease, and neurodegenerative disease [24,25]. To further evaluate the status of autophagy flux in MDA-MB-231 cells after CLE-10 treatment, a tandem mRFP-GFP-LC3 adenovirus was applied. The increasing number of green GFP-LC3 dots and red mRFP-LC3 puncta illustrated the existence of autophagy. Merged images indicated that CLE-10 increased autolysosomes more than autophagosomes, thus stimulating autophagic flux.

Generally, it is uncertain whether autophagy serves as a cell death or survival mechanism, or a bystander in dying cells [26]. In order to study the roles of autophagy induced by CLE-10 in MDA-MB-231 cells, CLE-10 was pretreated with an autophagy inhibitor (3-MA, CQ) or a mTOR inhibitor rapamycin. Autophagy inhibitors 3-MA and CQ weakened the effect of CLE-10 on the growth of MDA-MB-231 cells, while rapamycin enhanced the cytotoxicity of CLE-10, with the IC_50_ value decreasing from 4.07 µM to 2.38 µM. The results revealed that autophagy induced by CLE-10 in MDA-MB-231 cells contributed to cell death rather than promoted cell survival.

Accumulated evidence indicated that apoptosis and autophagy could be induced via the same upstream signals that affect the occurrence, proliferation, and treatment of cancer, such as PI3K/Akt/mTOR, p53, and Bcl-2 signaling pathways [27,28]. PI3K/Akt/mTOR are significant kinases activated by various stimuli. They regulate essential cellular functions including proliferation, transcription, translation, growth, and survival. PI3K, Akt, mTOR, and autophagy-related protein (ULK1, LC3, p62) expressions were analyzed in order to confirm whether this pathway was involved in CLE-10-induced cell death. CLE-10 treatment for 12 h or 24 h in MDA-MB-231 cells downregulated p-PI3K, p-Akt, p-mTOR, p62, and LC3-Ⅰand increased the protein expression of p-ULK1 and LC3-Ⅱ in a dose-dependent manner, but did not exert a notable impact on the expression of these total proteins, thus suggesting that CLE-10 inhibited the PI3K/Akt/mTOR signal pathway in MDA-MB-231 cells.

Moreover, mTOR, ULK1, LC3, and p62 are involved in the process of autophagosome formation or degradation. The formation of the Atg1/ULK1/ATG13/FIP200 protein-kinase complex is essential in the initiation of phagophore formation. Meanwhile, ULK1 is activated by decreased mTORC1 signaling or increased AMPK activity, leading to the phosphorylation of ATG13 and FIP200 [29]. There are three types of LC3 present in the cell: pro-LC3, LC3-I, and LC3-II. LC3-I protein continues to exist in the cytoplasm, while LC3-II integrates into both sides of the membrane, forming autophagosomes, and LC3-II is degraded with the membrane by lysosomal enzymes [30]. The level of LC3-II is related to the amount of autophagosomes in the cell; thus, LC3-II acts as a reliable marker of autophagy [31]. p62, a traditional receptor of autophagy, is involved in ubiquitinated cargoes delivering autophagic degradation [32]. Moreover, the decreasing expression of p62 activates autophagy and the deletion of p62 leads to LC3-II formation, aggresome or autophagosome impairment, cell damage, and finally cell death [33].

It is significant that the expression of mTOR, ULK1, LC3-II, and p62 in the autophagy process was detected with or without 3-MA and CQ. When CLE-10 was used to treat MDA-MB-231 cells alone, the increasing expression of LC3-II and p-ULK1 and the decreasing p62 expression were detected compared to the 0 µm CLE-10 group, suggesting that CLE-10 induced autophagy. Meanwhile the CLE-10 + 3-MA group weakened the upregulation of protein expression of ULK1 and LC3-II or the downregulation of p62 compared to the CLE-10 group. The CLE-10 + CQ group increased the protein expression of LC3-II compared to the CLE-10 group, indicating that CLE-10 increased the formation of autophagosomes. In general, CLE-10 exhibited a similar effect on the autophagy-related proteins as rapamycin and the influence of CLE-10 on the autophagy-related proteins could be weakened by 3-MA or CQ.

## 4. Material and Methods

### 4.1. Materials

CLE-10 was isolated from CAL, as mentioned in our previous article [20]. The purified CLE-10 (over 99% pure) was dissolved in dimethylsulfoxide (DMSO) as a 25 mM solution and stored at 4 °C. Once used, the CLE-10 solution was diluted with a culture medium to a desired concentration.

### 4.2. Cell Lines and Cultures

The human cervical carcinoma Caski, human mammary carcinoma cell line MDA-MB-231, human lung cancer cell line A549, human colorectal carcinoma cell line CaCo-2, human nasopharyngeal carcinoma CNE-2, human SH-SY5Y neuroblastoma cell line, human hepatocellular carcinoma HepG-2 cell lines, human gastric carcinoma HGC-27 cell lines, monkey embryonic renal epithelial cell line (Marc-145), human gastric cell line (GES-1), and Madin–Darby canine kidney cell line (MDCK) were bought from China Type Culture Collection in Shanghai and preserved in our laboratory. Cells were cultured in RPMI-1640, L-15, or DMEM culture medium with 10% fetal bovine serum at 37 °C in 5% CO_2_.

### 4.3. MTT Assays

One hundred microliters of the cell suspension diluted with culture medium (0.8–1 × 10^5^ cell/mL) were added to the 96-well microplates. Twelve hours later, 100 µL of culture medium with different concentrations of CLE-10 from 3.12 µM to 100 µM were added to each well, which were incubated for another 48 h. Then, 20 µL of 5 mg/mL MTT (Sigma-Aldrich, Shanghai, China) reagent was added per well. After culturing the cells for 4 h, they were gently removed the medium and then 150 µL DMSO solution (Sigma-Aldrich) was added to each well. The absorbance was detected by a microplate reader (Tecan Shanghai, China) at 490 nm, subtracting the baseline reading.

### 4.4. CLE-10 Pretreated with 3-MA, CQ, Rapamycin Using MTT Assays

Cells (8–9 × 10^4^/mL) were seeded into 96-wel plates in 100 µL of medium and were cultured for 12 h. The cells were divided into five groups. Cells treated with normal culture media were regarded as the control group. For the CLE-10 group, cells were treated with medium containing CLE-10 at various concentrations (3.12–100 µM). For inhibitor groups, cells were pretreated with a selective autophagy inhibitor (3-MA (5 mM), CQ (20 µM)) or the mTOR agonist rapamycin (100 nM) for 6 h, then the supernatant was gently removed and replaced by culture medium containing CLE-10 at various concentrations (3.12–100 µM) for 48 h. Next, 20 µL of 5 mg/mL MTT was added to all the wells for another 4 h. The medium was gently removed, after which 150 µL of DMSO was added to each well. The absorbance of each well was detected by a microplate reader at 490 nm by subtracting the baseline reading. Autophagy inhibitors 3-methyladenine (3-MA) and chloroquine (CQ) were obtained from MCE China, as was the mTOR inhibitor (rapamycin).

### 4.5. Flow Cytometry (FCM)

Apoptosis was detected with an Annexin V-Enzo Gold apoptosis detection kit (Enzo Life Sciences, Beijing, China). MDA-MB-231 cells were treated with CLE-10 (0, 10, 15 µM) for 24 h. Cells were gathered by trypsinization and washed using cold phosphate-buffered saline (PBS) twice. Then cells were resuspended in buffer or buffer containing Annexin V-EnzoGold or Necrosis Detection Reagent according to the instruction at room temperature, avoiding light for 15 min, and analyzed by flow cytometry (BD Bioscience).

### 4.6. Western Blot Analysis

MDA-MB-231 cells were gathered and the total proteins in each sample was obtained and quantified using the bicinchoninic acid (BCA) protein concentration assay kit (Biyuntian, Beijing, China) after treatment with different concentrations of CLE-10 (0, 5, 10, 15 µM) for 24 h. Equal amounts of protein (50 µg) was separated by 6–15% SDS-PAGE gels and transferred to polyvinylidene fluoride (PVDF) membranes (Beijing Labgic Technology Co., Ltd.). Membranes were blocked with 5% milk (BD) for 2 h. After washing, the membranes were probed overnight using the specific primary antibodies LC3, PI3K (p85), AKT, mTOR, p-PI3K (Tyr 508), p-AKT (Ser473), p-mTOR, p-ULK1(Ser 757), p62, Bax, Bad, Bcl-2, and Bcl-xl (Cell Signaling Technology) at 4 °C, followed by secondary antibody. Chemiluminescence was performed with a chemiluminescence developing solution (Biyuntian, Beijing, China) on Kodak X-ray films or using a chemiluminescence image analysis system (Tanon 5200). LC3, p-ULK1, ULk1, p62, mTOR, and p-mTOR were also detected with or without an autophagy inhibitor (CQ, 3-MA) or the mTOR agonist rapamycin. Cells were treated with 3-MA (5 mM), CQ (40 µM), or rapamycin (100 nm), CLE-10 (15 µM), 3-MA (5 mM) + CLE-10 (10 µM), CQ (40 µM) + CLE-10 (15 µM), and rapamycin (100 nm) + CLE-10 (15 µM); each for 24 h. Western blot analysis was used to detect the relative protein expression as described above.

### 4.7. Transmission Electron Microscopy (TEM)

For TEM analysis, CLE-10 (0, 15 µM) was applied for 24 h, after which MDA-MB-231 cells were gathered and fixed in ice-cold 2.5% glutaraldehyde containing 0.1 M cacodylate buffer with a pH of 7.4. In 1% phosphate-buffered osmium tetroxide, cells were subsequently stained with 3% aqueous uranyl acetate. Cells were then dehydrated in an increasing gradient of ethanol solution and embedded in epoxy resin. Ultrathin sections were gained and later stained. Electron micrographs were observed and imaged using a transmission electron microscope (H7650; Hitachi, Tokyo, Japan).

### 4.8. Tandem mRFP-GFP-LC3 Transfection

A tandem mRFP-GFP-LC3 adenovirus (Hanheng Biotechnology Co Ltd., Shanghai, China) was transfected into incubated MDA-MB-231 cells in a culture dish (Wuxi NEST Biotechnology, China) for 6.5 h at a multiplicity of infection (MOI) of 200 before receiving CLE-10 (30 µM) treatments. After treating for 24 h, cell images were obtained by laser confocal fluorescence microscopy (LCFM) (Olympus FV1200, Japan).

### 4.9. Statistical Analysis

The data of all the experiments were described as means ± SD. Statistical analyses were determined using the Graphpad prism 5.0 statistical software. Statistical differences were evaluated using unpaired the Student’s *t*-test and the ANOVA method, and were considered to be significantly different at *p* < 0.05.

## 5. Conclusions

In this study, our results showed that CLE-10 significantly suppressed the growth of human breast cancer cells (MDA-MB-231). Furthermore, CLE-10 affected many autophagy- and apoptosis-related proteins including p-ULK1, LC3-II, p62, Bax, Bad, Bcl-2, and Bcl-xl, and suppressed the PI3K/AKT/mTOR pathway, ultimately inducing apoptosis and pro-death autophagy in MDA-MB-231 cells. In addition, autophagy inhibitors (3-MA or CQ) weakened while rapamycin enhanced the effect of CLE-10 on the autophagy-related proteins in addition to the cytotoxicity of CLE-10 against MDA-MB-231 cells. Our results revealed a potential mechanism of CLE-10-induced cell death, providing a guide for the use of CLE-10 in in vitro studies.
